# Oxidation mechanism of T91 steel in liquid lead-bismuth eutectic: with consideration of internal oxidation

**DOI:** 10.1038/srep35268

**Published:** 2016-10-13

**Authors:** Zhongfei Ye, Pei Wang, Hong Dong, Dianzhong Li, Yutuo Zhang, Yiyi Li

**Affiliations:** 1 Shenyang National Laboratory for Materials Science, Institute of Metal Research, Chinese Academy of Sciences, 72 Wenhua Road, Shenyang, 110016, China; 2 Shenyang Ligong University, 6 Nanping Road, Shenyang, 110159, China

## Abstract

Clarification of the microscopic events that occur during oxidation is of great importance for understanding and consequently controlling the oxidation process. In this study the oxidation product formed on T91 ferritic/martensitic steel in oxygen saturated liquid lead-bismuth eutectic (LBE) at 823 K was characterized at the nanoscale using focused-ion beam and transmission electron microscope. An internal oxidation zone (IOZ) under the duplex oxide scale has been confirmed and characterized systematically. Through the microscopic characterization of the IOZ and the inner oxide layer, the micron-scale and nano-scale diffusion of Cr during the oxidation in LBE has been determined for the first time. The micron-scale diffusion of Cr ensures the continuous advancement of IOZ and inner oxide layer, and nano-scale diffusion of Cr gives rise to the typical appearance of the IOZ. Finally, a refined oxidation mechanism including the internal oxidation and the transformation of IOZ to inner oxide layer is proposed based on the discussion. The proposed oxidation mechanism succeeds in bridging the gap between the existing models and experimental observations.

Ferritic/martensitic (F/M) steels are the preferred structural material for advanced energy generating systems, such as advanced nuclear systems^1^ and ultra-supercritical coal-fired power-generating units (USC)^2^,^3^. For various reasons, these in-service F/M steels inevitably face one common issue: oxidation. For instance, the F/M steels serving in USC and super-critical water-cooled reactors suffer severe oxidation from the water medium (supercritical water or steam) at high temperature^4^,^5^,^6^. And the F/M steels in accelerator-driven systems are deliberately oxidized to prevent the liquid metal corrosion by lead-bismuth eutectic (LBE)^7^,^8^,^9^.

Coincidentally, the oxidation behaviors of F/M steels, in CO_2_^10^,^11^, water^4^,^5^,^12^, steam^13^,^14^, and LBE^15^,^16^ are very similar. All the oxidation processes lead to the formation of a duplex oxide scale composed of a magnetite outer layer and a Cr enriched Fe-Cr spinel inner layer^17^,^18^,^19^,^20^. Given the similarities, analogous models have been proposed to interpret the oxidation phenomena in these oxidants^8^,^21^.

Among the models established to interpret the bilayer structure^22^,^23^,^24^,^25^, the “available space model” proposed by Bruckman and Romanski in 1965 has been accepted as the most successful one^25^. The model indicates that Fe atoms diffuse outwards to form an outer oxide scale at the oxide-oxidant interface, leaving abundant vacancies behind. The vacancies condense at the oxide scale-metal interface into voids, leading to the dissociation of the oxide scale above these voids, which produces nano/micro-channels in the oxide scale. Once the nano/micro-channels emerge, the oxidant diffuses through them to the metal/oxide interface to form a new inner oxide scale, i.e., the inner oxide scale grows into the metal from the metal/oxide interface. Because the transportation of oxygen through the nano/micro-channels is significantly fast to be the growth-rate-controlling step for the inner oxide layer, the available space created by the outward diffusion of Fe is proposed to determine the growth rate of the inner layer in the model. This is why this model is called the “available space model”. The details of the model can be found in refs 25 and 26. On the basis of “available space model”, several optimized models have been proposed to more accurately interpret or even predict the evolution process of duplex oxide layer structures^9^,^27^,^28^,^29^.

In addition to the duplex oxide layers, a layer of oxygen diffusion zone (ODZ) often appears beneath the inner oxide layer^30^,^31^,^32^. In contrast to the outer and inner oxide layers, the ODZ has received little attention either experimentally or theoretically. However, as the oxidization front and the origin of the inner oxide layer, the importance of the ODZ is sufficiently evident.

Moreover, though Cr is an important element that influences the oxidation properties, it has not drawn enough attention during oxidation of F/M steels in special oxidizing media (such as, supercritical water, steam, CO_2_, and LBE). The diffusion of Cr in the oxide layer is said to be negligible in the “available space model” because of the remarkable difference between the diffusion coefficients of Fe and Cr in the spinel lattice; therefore, the concentration of Cr in the inner oxide layer has been proposed to be a constant^33^. Meanwhile, little focus has been put on the behavior of Cr in the matrix during oxidation. In view of the significant role of Cr in enhancing the oxidation resistance of F/M steels^34^, much more attention should be paid to the behavior of Cr during oxidation of F/M steels in special oxidizing media.

In summary, the current oxidation models, such as the “available space model”, predict the oxidation kinetics successfully and give rational explanations for the observed structure of oxide scales. However, the in-depth microscale mechanism that dominates the oxidation, especially the origination of oxidation, is still unknown. In order to explore the underlying mechanism to better understand and control the oxidation of F/M steels in special oxidizing media, the oxidation of T91 in oxygen saturated LBE at 823K was performed in this work. Significant efforts have been made to characterize and analyze the reaction occurring in the oxidation front, i.e. the ODZ, at nanoscale, with the purpose of comprehending the origin and evolution of the oxidation. Additionally, the occurrence of diffusion of Cr during oxidation and its importance have been determined unambiguously for the first time in this study. Finally, based on the microscopy analyses, a refined oxidation model involving the formation and evolution of the ODZ is proposed to illuminate the mechanism accounting for the oxidation of T91 in oxygen saturated liquid LBE.

## Results

### A glance at the oxidized cross section

**Figure 1:**
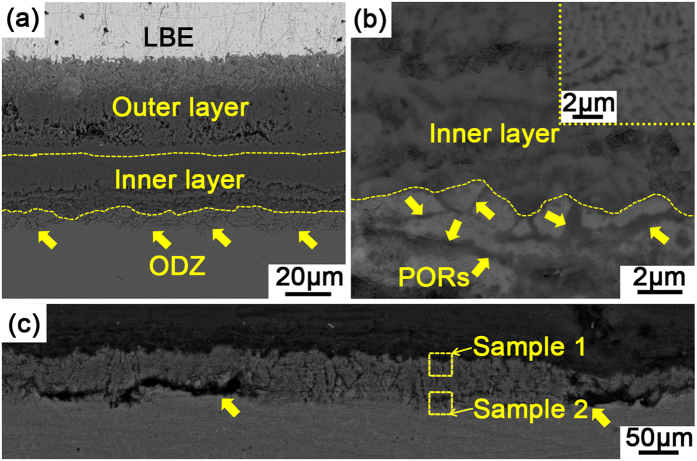
Cross-section back-scattered electron image of the oxide scale formed on T91 steel after being exposed in oxygen saturated LBE at 823 K for 2000 h. (**a**) The whole cross-section image. The arrows indicate the ODZ. (**b**) Morphology of the ODZ beneath the inner oxide layer. The inset picture shows that many oxide spots exist in the “matrix” surrounded by the PORs. The arrows indicate the PORs in ODZ. (**c**) The sites for TEM samples preparation. The arrows indicate Cr_2_O_3_.

Consistent with the literature^4^,^9^,^15^, the oxide scale formed on T91 steel after being exposed in oxygen saturated LBE at 823 K for 2000 h is composed of two layers (see Fig. 1a). Beneath the duplex oxide scale, there is a layer of oxygen diffusion zone. The outer oxide layer is porous and composed of columnar grains, with some penetration of LBE in the layer. The inner layer can be roughly divided into two sub-layers by their different morphologies. The sub-layer immediately beneath the outer oxide layer is compact, while the one above the ODZ is much more porous.

The ODZ beneath the duplex oxide scale is composed of two regions with very different morphology, i.e., the preferential oxidization regions (PORs) as indicated by the arrows in Fig. 1b and the “matrix” surrounded by the PORs (see Fig. 1b). The PORs in the ODZ are mainly located at the grain/subgrain boundaries. Careful observation shows that many oxide spots exist in the “matrix” surrounded by the PORs (see inset of Fig. 1b). The similar morphology has also been reported in refs 30 and 31 in 9–12% Cr F/M steel oxidized in supercritical water and steam oxidation environment. It is obvious that the ODZ is the origin of inner oxide layer. Therefore, characterization and analysis of the microstructure and elements distribution in ODZ are of great importance.

### Identification of ODZ

In order to clarify the microstructure of ODZ, especially the small oxide spots in the “matrix” surrounded by PORs, transmission electron microscope (TEM) specimens were “lifted out” from the ODZ using focused ion beam (FIB) for characterization. The locations of the TEM samples are indicated approximately by the dotted boxes in Fig. 1c. *Sample 1* and *sample 2* are located at the inner oxide layer/ODZ interface and the ODZ/metal interface, respectively. The sites for TEM sample preparation were chosen with the purpose of investigating the dynamic evolution process of the ODZ, i.e., the transformation from the martensitic matrix to the ODZ (*sample 2*) and the evolution from the ODZ to the inner oxide layer (*sample 1*).

**Figure 2:**
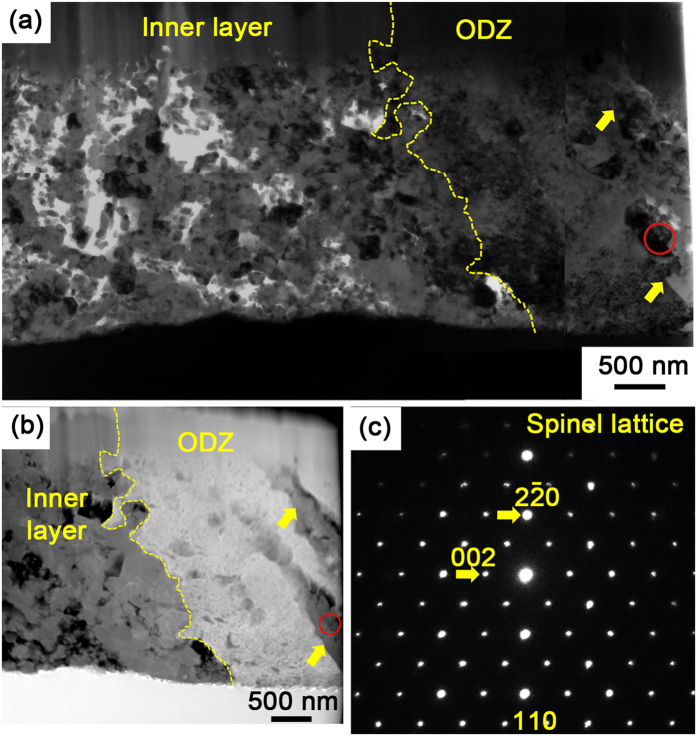
The morphology of the interface between the inner oxide layer and ODZ. (**a**) Bright-field TEM image of *sample 1* indicated in 1(c). The arrows indicate PORs. The red circle indicates the SAED site. (**b**) Corresponding HAADF-STEM image taken around the interface indicated by dotted line in (**a**). The arrows indicate PORs. The red circle indicates the SAED site. (**c**) The SAED pattern acquired from the PORs indicated by circles in (**a,b**).

Figure 2a shows the bright field TEM image of *sample 1.* Figure 2b is the corresponding high angle annular dark field-scanning transmission electron microscope (HAADF-STEM) image taken around the interface indicated by the dotted line in Fig. 2a, which provides additional information on Z-contrasts to distinguish the oxidized regions more conveniently. The PORs are indicated by the arrows in Fig. 2a,b. The corresponding selected area electron diffraction (SAED) pattern in Fig. 2c reveals that the PORs have a spinel structure (the circles in Fig. 2a,b indicate the site at which the SAED pattern was acquired).

**Figure 3:**
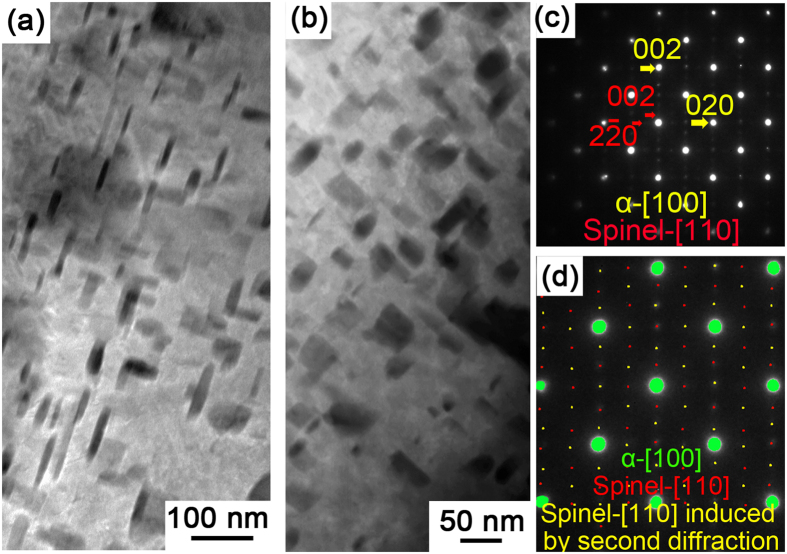
The morphology and microstructure of the lath interior surrounded by PORs in ODZ. (**a**) The morphology of the oxide precipitates in lath interior surround by PORs in sample 1; (**b**) The morphology of the oxide precipitates in lath interior surround by PORs in sample 2; (**c**) The SAED pattern taken from the lath interior surround by PORs; (**d**) Detailed analysis of the SEAD pattern in (**c**).

Figures 3a,c show the morphology and the corresponding SAED pattern of the lath interior surrounded by PORs. The SAED pattern demonstrates that the small oxide precipitates also have a spinel structure and that the matrix preserves the pristine b.c.c Fe structure. The small spinel particles and the surrounding b.c.c matrix have a Baker-Nutting relationship, i.e. {001}_spinel_//{001}_matrix_ and <100> _spinel_//<110>_matrix_, which is also observed by Bischoff *et al.*^31^ in HCM12A steel that oxidized in 873K supercritical water. Detailed SAED pattern analysis for Fig. 3c is shown in Fig. 3d and Supplementary Note 1. As shown in Fig. 3a,b, along with the oxidation time increasing, the aspect ratio of the oxide precipitates increases, i.e. the particle transforms from nearly square to rectangular. In addition, the oxide precipitates are distributed regularly. The reasons accounting for the morphology evolution of oxide precipitates are discussed in Supplementary Note 2.

### Inhomogeneous Cr distribution in the oxide product

**Figure 4:**
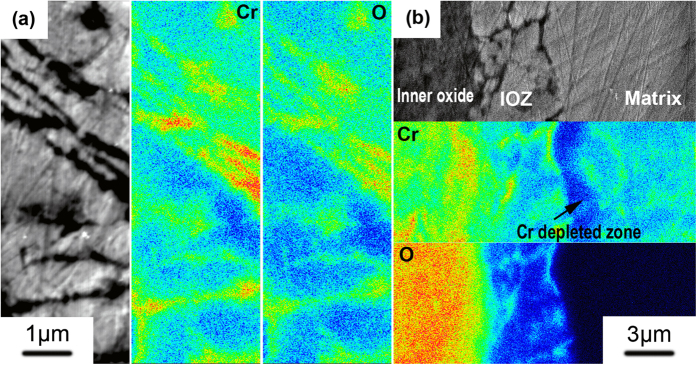
The inhomogeneous elements distribution in ODZ and Cr-depleted zone beneath the ODZ. (**a**) EPMA characterization showing the Cr and O distribution in ODZ; (**b**) Cr depleted zone beneath the ODZ identified by EPMA.

**Figure 5:**
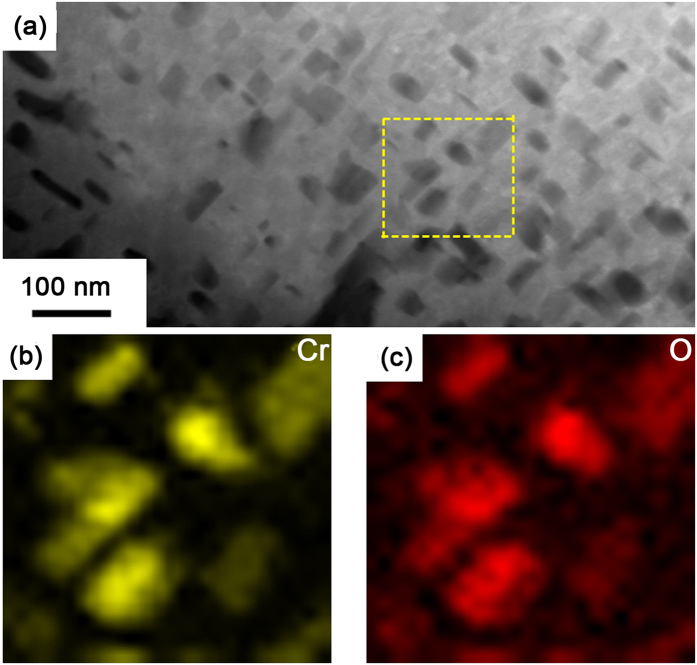
The morphology and elements distribution of the oxide precipitates in lath interior surrounded by PORs in ODZ. (**a**) Morphology of the oxide precipitates; (**b**) EDS mapping scanning showing the Cr distribution in the framed area indicated in (**a**); (**c**) EDS mapping scanning showing the O distribution in the framed area indicated in (**a**).

Electron probe micro-analyzer (EPMA) characterization shows that the chemical concentration of Cr is not homogeneous at the micrometer scale in the ODZ. The PORs in the ODZ are significantly enriched with Cr, while Cr depletion is evident in the regions surrounded by these PORs (see Fig. 4a). Much more refined chemical analysis of the oxide precipitates in the ODZ was performed by electron dispersive spectroscopy (EDS) in the TEM. Figure 5b reveals the Cr distribution measured by EDS mapping scanning in the framed area indicated in Fig. 5a. The enrichment of Cr in these oxide precipitates indicates the inhomogeneous distribution of Cr at the nanometer scale in the ODZ. In addition to oxide precipitates, the chemical composition of the PORs was also measured by EDS in the TEM. Consistent with EPMA results, all the PORs are enriched with Cr. Bischoff 30,31 *et al.* also reported the enrichment of Cr in PORs that along the lath/grain boundaries and in the oxide precipitates in lath interior, which is consistent with our observation. Beneath the ODZ there is a Cr-depleted zone in the matrix (see Fig. 4b), which has not received sufficient attention in previous studies. Benamati *et al.* mentioned in ref. 35 that depletion of Cr occurred in the region under the steel/reaction products interface, which is similar to our results; however, they did not provide a rational explanation or discussion on this phenomenon.

**Figure 6:**
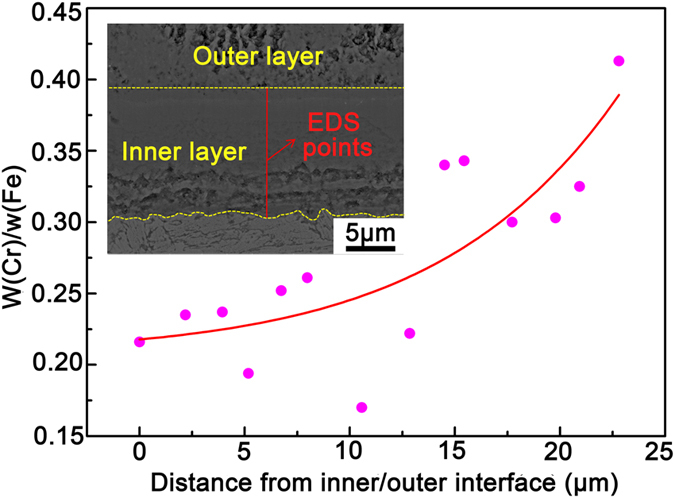
Variation of w(Cr)/w(Fe) along the thickness direction of inner oxide layer. The value of w(Cr)/w(Fe) increases from the inner/outer oxide layer interface to the inner oxide layer/ODZ interface.

Except for the inhomogeneous distribution of Cr at the micrometer and nanometer scales in the ODZ, the Cr concentration does not remain constant along the thickness direction in the inner oxide layer. The value of w(Cr)/w(Fe) increases gradually from the inner/outer oxide layer interface to the inner oxide layer/ODZ interface, as shown in Fig. 6 (the EDS points are indicated in the inset of Fig. 6). It is contradictory to the hypothesis in the “available space model” and the reported results in refs 8, 16 and 33.

## Discussion

The morphology of the ODZ involves dispersive oxide particles and oxidized grain/subgrain boundaries in a metal matrix. According to the definition of internal oxidation, i.e., a process in which oxygen diffuses into an alloy and causes sub-surface precipitation of the oxides of one or more alloying elements^36^, the ODZ demonstrates typical characteristics of internal oxidation. It is therefore indicated that an internal oxidation zone (IOZ) exists beneath the inner oxide layer.

Once the internal oxidation occurs, the continuous advancement of IOZ becomes the essential process of the oxidation. One of the necessary conditions for the advancement of internal oxidation is that the solute concentration of the alloy must be lower than that required for the transition from internal to external oxidation. In the meantime, no oxide layer prevent the dissolution of oxygen into the alloy. In our experiment, as oxide scale thickness increases with oxidation time, the oxygen potential at the IOZ/matrix interface decreases gradually. According to the theory of high temperature oxidation, a decrease in oxygen potential leads to a decrease in the critical Cr content above which a continuous Cr_2_O_3_ layer forms^36^. Therefore, if the Cr concentration remains constant, a continuous Cr_2_O_3_, which can prevent oxygen from dissolving into the alloy, would form eventually and the internal oxidation would be interrupted. In reality, Cr_2_O_3_ is present at some sites of IOZ/matrix interface (see Supplementary Fig. 1 and indicating arrows in Fig. 1c). However, the decrease in the Cr concentration in the Cr-depleted zone prevents the formation of continuous Cr_2_O_3_ scale, and thus the oxygen is able to penetrate into matrix to sustain internal oxidation. Therefore, the Cr-depleted zone plays important role during the advancement of the IOZ and in turn the oxidation process.

EDS analysis of Cr-depleted zone in Supplementary Fig. 2 displays a gradual increase in the value of w(Cr)/w(Fe) from the IOZ/Cr-depleted zone interface to the Cr-depleted zone/matrix interface, showing the most obvious chemical distribution with diffusion character. In addition, the inhomogeneous distribution of Cr in the IOZ (see Figs 4a and 5) and inner oxide layer (see Fig. 6) also demonstrate unambiguously the non-negligible diffusion of Cr during the oxidation process. However, to the best of our knowledge, nearly all the literature concerning the formation process of a bilayer structure on Fe-Cr alloys during oxidation seemed to neglect Cr diffusion^33^,^37^. Bischoff *et al.*^30^,^31^ have mentioned that once the oxygen diffuses into the metal grain an iron-chromium segregation occurs at nanometric scale to explain the appearance of nanometric chromium-rich spinel oxides in the grain interior in some 9–12% Cr steel oxidized in supercritical water and steam. And the Cr-rich PORs at the grain/lath boundaries are induced by the oxidation of Cr_23_C_6_ carbides. Here, we propose that the diffusion of Cr is accomplished in two ways based on our experimental evidence: I, long-range diffusion at the micron-scale; II, short-range diffusion at the nanoscale.

Long-range diffusion at the micron-scale accounts for the appearance of Cr-depleted zone. The oxidized regions (including PORs and oxide particles) in the IOZ are enriched with Cr compared to the T91 matrix, which means that the transportation of Cr atoms from the b.c.c Fe-Cr lattice to the oxide has taken place in IOZ. The transportation makes chemical potential of Cr in the b.c.c Fe-Cr lattice in the IOZ lower than that in the adjacent matrix, which results in the diffusion of Cr from the adjacent matrix to the IOZ and hence leads to the appearance of a Cr-depleted zone.

**Figure 7:**
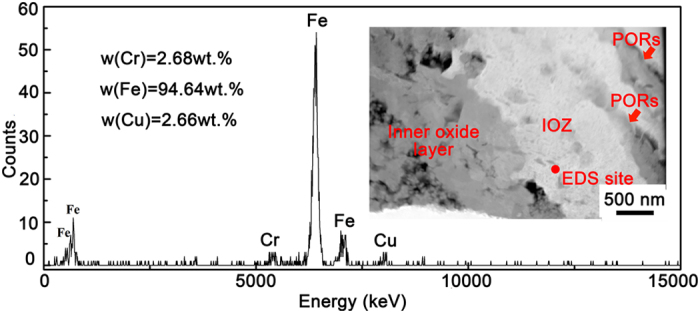
Semi-quantity EDS analysis of the lath interior surrounded by PORs.

Based on the experimental observations that PORs and oxide precipitates in IOZ are rich in Cr, we propose that the diffusion of Cr at the nanoscale occurs in two different ways, i.e., transportation from the lath interior to grain/subgrain boundaries, and repartition in the lath interior. The former is driven by the preferential oxidation. When the oxygen contacts metal matrix at the bottom of nano/micro-channels, it preferentially diffuses into the steel matrix through the fast diffusion pipe, i.e., grain/subgrain boundaries. The Cr_23_C_6_ carbides at the grain/lath boundaries are oxidized into the Cr-rich PORs^30^,^31^. In addition, the presence of oxygen at the boundaries results in the oxidation of metal atoms, especially Cr. The oxidation reaction consumes large amounts of Cr at the boundaries and leads to the significant decrease in chemical potential of Cr in the Fe-Cr lattice. In order to maintain chemical potential equilibrium, Cr diffuses from the lath interior to the lath boundaries. The diffusion leads to the depletion of Cr in the lath interior, which is confirmed by the measured ~2 wt.% Cr concentration in the lath interior surrounded by PORs (see Fig. 7, the signal of copper is from the Cu grid of the FIB sample). It is much lower the average Cr concentration in the experimental material.

Similar to the repartition of Cr in the lath interior, segregation of Cr has been observed in the IOZ during the oxidation of ferritic/martensitic steel in supercritical water and steam^30^,^31^ and austenitic stainless steel in LBE^38^. The authors in ref. 38 attributed the segregation to spinodal decomposition induced by the presence of oxygen. However, in consideration of the thermodynamic calculation (in Supplementary Note 3) we do not think spinodal decomposition can occur in our alloy. We propose that the presence of oxygen induces the short-range diffusion of Cr at the nanoscale^30^,^31^. The solubility of oxygen in the Fe-Cr lattice is too low for oxidation to occur at the beginning. Therefore, nano-clusters without specific structure enriched with oxygen may form first. Because Cr has better oxygen affinity than Fe, these clusters are rich in Cr. In the following stage, as the supplement of oxygen increases, these clusters gradually grow into Cr_2_O_3_ and/or spinel particles. Therefore, similar to that at the boundaries, the chemical potential of Cr in the b.c.c. Fe-Cr lattice around oxide nuclei decreases significantly. In order to maintain chemical potential equilibrium, diffusion of Cr from the surrounding matrix to these oxide nuclei occurs.

Diffusion of Cr at the nanoscale gives rise to the Cr-enriched PORs and the dispersed nanometer oxide precipitates in the IOZ. These pre-oxidized sites are the most typical characteristic of the IOZ and the evolution of these sites is of great importance to the transformation of the IOZ into an inner oxide layer, i.e. the process of oxidation.

**Figure 8:**
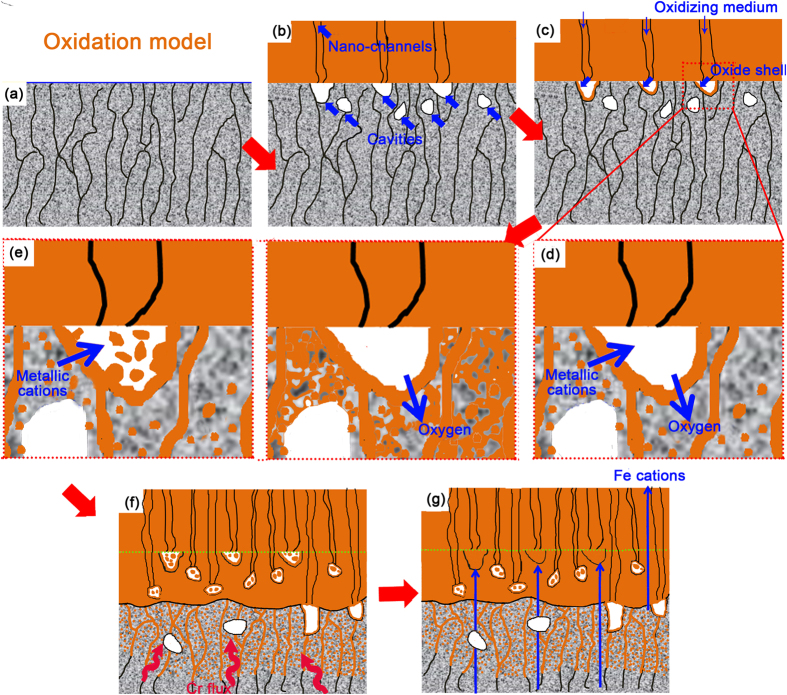
Schematic illustration of the proposed refined oxidation mechanism.

The above discussion demonstrates that internal oxidation and diffusion of Cr occur during oxidation of P91 in LBE. However, the existing oxidation models have not taken these two important phenomena into consideration. Therefore, a refined oxidation mechanism is proposed to bridge the gap between experimental observations and existing models. A sketch illustrating the oxidation process is shown in Fig. 8. When the steel contacts the oxygen-saturated liquid LBE, a thin oxide scale forms on the steel surface rapidly (see Fig. 8a). However, this thin oxide scale can not impede the outward diffusion of Fe cations effectively, which results in the formation of the outer layer. At the same time, the outward diffusion of Fe gives rise to large amounts of vacancies in the steel matrix and these vacancies accumulate into cavities (see Fig. 8b). Given the growth kinetics of the oxide layer, nano-channels are proposed to serve as the fast diffusion path of oxygen, which is similar to that of the “available space model”. The nano-channels are proposed to form in a dissociative/perforative manner^39^, i.e. nano-channels nucleate by dissociation of the oxide grains above cavities (see Fig. 8c). The oxidizing agent in the nano-channel is in equilibrium with the surrounding oxide scale^39^. Thus, there is a gradient in the chemical potential of oxygen in the nano-channels along the direction of thickness in the oxide scale.

As soon as the oxygen in the nano-channels meets the steel matrix, oxidation occurs, and oxide shells form in the inner walls of the cavities (see Fig. 8c) firstly. Once the oxide shell forms, oxidation can proceed by the diffusion of oxygen through the oxide shell to the steel matrix and/or by the counter diffusion of metal cations (mainly Fe cations) through the oxide shell to the cavities (see Fig. 8d). The diffusion of oxygen through oxide shell into the steel matrix results in the typical internal oxidation character.

Once the IOZ appears, evolution from the IOZ to the inner oxide layer becomes the key process to maintain the continuous advancement of the oxide scale into the steel matrix. The process of transformation from the IOZ to the inner oxide layer actually includes phase homogenization and chemical homogenization.

Phase homogenization is accomplished in two different ways as shown in Fig. 8e. The first one is induced by the diffusion of metal cations from the steel matrix through the oxide shell to the cavities. Because of the higher oxygen potential in the cavities, the metal cations are oxidized in the cavities *in situ*. The other one is induced by the diffusion and accumulation of oxygen in the un-oxidized region. At the early stage of the IOZ, the oxygen potential is too low to oxidize the Cr-depleted regions adjacent to oxide precipitates. As the oxidation time is extended, oxygen is transported continually to the IOZ and accumulates there. At the same time, Cr diffuses from the matrix to the Cr depleted regions in IOZ. Therefore, the chemical potential of O and Cr in IOZ increase simultaneously gradually, making the oxidation possible in Cr depleted regions.

**Figure 9:**
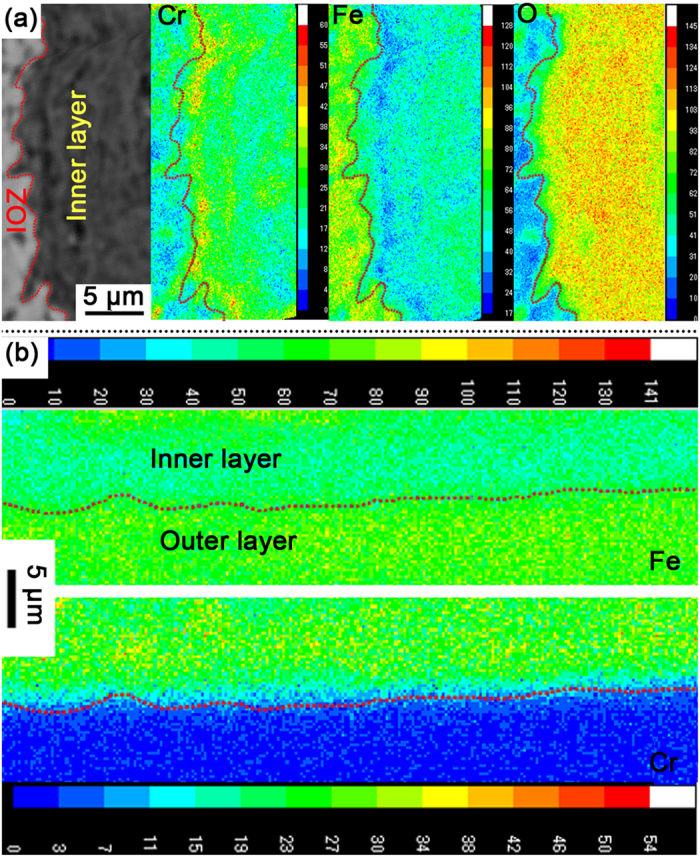
The chemical distribution in different sub-layers of inner oxide layer. (**a**) Chemical distributions in the inner layer adjacent to IOZ; (**b**) Chemical distributions in the inner layer adjacent to outer oxide layer.

Chemical homogenization, which is a much slower process than phase homogenization, proceeds continuously during and after the phase homogenization process. Figure 9a shows the Fe, Cr and O distributions at the lower sub-layer of inner oxide layer. Both Fe and Cr show clear chemical inhomogeneity. However, the distribution of oxygen in the same region is much more homogeneous, indicating that phase homogenization has been already accomplished. After sufficient time for diffusion, both chemical and phase homogenization are completed and a sound spinel layer with a homogeneous chemical distribution appears in the inner layer adjacent to the outer layer as shown in Fig. 9b.

As schematically shown in Fig. 8f, the inner oxide layer freshly transformed from IOZ is not perfectly compact, because of the oxide shell the cavities are not filled with oxide immediately. This results in the porous morphology of the lower sub-layer of the inner oxide layer as shown in Figs 1a and 2a. When the oxidation duration is extended, a portion of Fe cations diffusing outward are oxidized in the cavities surrounded by the oxide shell (see Fig. 8g). As the cavities are gradually filled by the oxide product, the inner oxide layer becomes much more compact (see Fig. 1a). The longer the oxidation time the more cavities are filled. Thus, the upper sub-layer of the inner oxide layer has a much more compact appearance. Because the cavities are filled by the oxidation product of Fe, the upper sub-layer of the inner oxide layer has a lower w(Cr)/w(Fe) value than the lower sub-layer. The measured w(Cr)/w(Fe) as a function of the distance from the outer/inner oxide layer interface shown in Fig. 6 demonstrates this argument.

According to the above analysis of the oxidation process, it can be found that the Cr-rich oxide, which can slow down the diffusion of oxygen and Fe/Cr cation, in the IOZ plays important role in the oxidation. Therefore, increasing the amount of Cr-rich oxide by increasing the Cr concentration in the bulk alloy can improve the oxidation resistance of the material. We have observed the oxidation behavior of some other 9–12% Cr ferritic/martensitic steels reported in refs 4, 5, 6, 7, 9, 10, 11, 12, 14, 15, 16, 17, 18 in different oxidant environment at the temperature ranging from 723K to 873K, all of them obey the above oxidation mechanism. However, if the Cr concentration in bulk alloy increases further, the internal oxidation will be interrupted by a continuous Cr_2_O_3_ forming at the oxidation front, in which the oxidation mechanism will be changed, such as the oxidation behavior of some austenitic steels described in refs 7, 15, 16, 17. In fact, except for the chemical composition, some methods that can accelerate the diffusion of Cr to form an effective Cr-rich oxide at the oxidation front also can improve the oxidation resistance of the alloy. We have reported that cold rolling enhances the oxidation resistance of T91 in LBE by introducing non-equilibrium grain boundaries and migrating dislocations into alloy, which accelerates the diffusion of Cr during the oxidation^40^.

## Conclusion

Through the systematical characterization on the microstructure of the oxidation product on T91 steel in oxygen saturated liquid LBE at 823K, we confirm internal oxidation occurs during the oxidation front, which induces an IOZ under the duplex oxide scale. During the oxidation process, the diffusion of Cr occurs at micro-scale and nano-scale. The micron-scale diffusion of Cr ensures the continuous advancement of IOZ and inner oxide layer, and nano-scale diffusion of Cr gives rise to the typical appearance of the IOZ.

## Methods

### Material and oxidation conditions

A laboratory T91 steel, with the composition of Fe-0.13C-7.95Cr-0.41Si-0.47Mn-0.83Mo-0.2V-0.1Nb-0.039N (wt.%) was prepared by vacuum induction melting. Hot forging at 1273–1423 K was carried out after casting to eliminate micro-segregation. The forging was normalized at 1323 K and tempered at 1033 K to obtain the tempered lathy martensitic microstructure (see Supplementary Fig. 3).

After heat treatments, specimens in a particular shape were cut from the forging, polished with diamond paste, and then ultrasonically rinsed in ethanol before oxidation. The oxidation experiment in LBE was carried out in a self-assembled equipment at 823 K with a duration up to 2000 h. A schematic illustration and photograph of the oxidation setup and the specimen for the oxidation experiment are shown in Supplementary Fig. 4. The liquid LBE was oxygen saturated during the entire oxidation process as indicated by the presence of red PbO particles on the liquid metal surface.

### Sample preparation and characterization

After the oxidation experiment, the specimens were mounted in ethoxyline directly without removing the LBE and polished for cross-sectional observations. A scanning electron microscope (SEM) (FEI Inspect F50) equipped with an energy dispersive spectrometer (EDS) (INCA X-Max) was used to perform the cross-sectional observation. The element distribution across the cross section was determined by a high resolution electron probe micro-analyzer (EPMA) (SHIMADZU EPMA-1610).

TEM samples were prepared using a FEI Helios Nanolab 650 Dualbeam, focus ion beam (FIB) system, with the method termed as *in-situ* lift-out (INLO)^41^. FIB was used instead of traditional mechanical thinning with ion milling, primarily because FIB allows for the production of site-specific specimens. After the sample was cut and mounted on a Cu grid, a 300 kV scanning transmission electron microscope (STEM) (FEI Tecnai F30 G2) with an EDS detector was used to characterize the microstructures. The STEM is equipped with a high angle annular dark field (HAADF) detector for Z-contrast imaging.

## Additional Information

**How to cite this article**: Ye, Z. *et al.* Oxidation mechanism of T91 steel in liquid lead-bismuth eutectic: with consideration of internal oxidation. *Sci. Rep.* **6**, 35268; doi: 10.1038/srep35268 (2016).

## Supplementary Material

Supplementary Information
