# Supplementary material for: Oxidation mechanism of T91 steel in liquid lead-bismuth eutectic: with consideration of internal oxidation
Source: Sci Rep. 2016 Oct 13;6:35268. doi: 10.1038/srep35268 (PMC5062345; doi:10.1038/srep35268)
Supplement: Supplementary Information [file srep35268-s1.pdf]

Supplementary File of the manuscript "Oxidation mechanism of T91 steel in liquid lead-bismuth eutectic: with consideration of internal oxidation"

Zhongfei Ye[a], Pei Wang[a*], Hong Dong[a], Dianzhong Li[a], Yutuo Zhang[b], Yiyi Li[a]

[a]Shenyang National Laboratory for Materials Science,

Institute of Metal Research, Chinese Academy of Sciences,

72 Wenhua Road, Shenyang, 110016, China

[b]Shenyang Ligong University, 6 Nanping Road, Shenyang, 110159, China

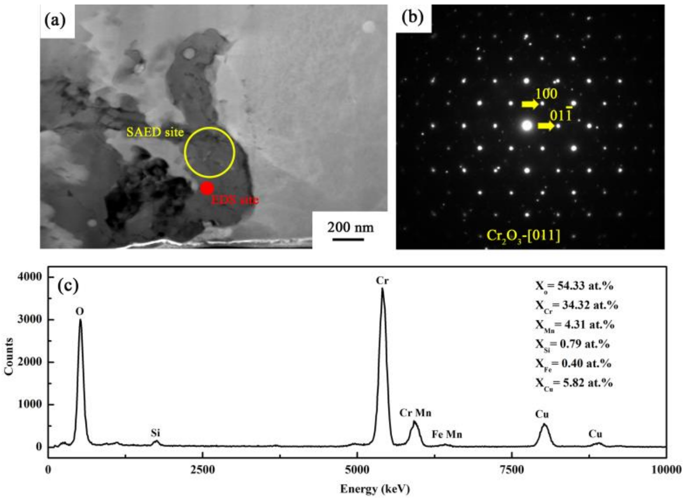

Supplementary Figure 1 | There are some Cr_2O_3 scale at IOZ/Cr depleted zone interface. (a) Typical morphology of Cr_2O_3 scale locating at IOZ/Cr depleted zone interface; (b) The SAED pattern acquired from the region indicated by yellow circle in (a); (c) EDS analysis of the region indicated by red point in (a) (The Cu signal arises from the contamination from the specimen holder)

*Corresponding author. Tel.: +86-24 83970106; fax: +86-24 83970097.
E-mail address: pwang@imr.ac.cn

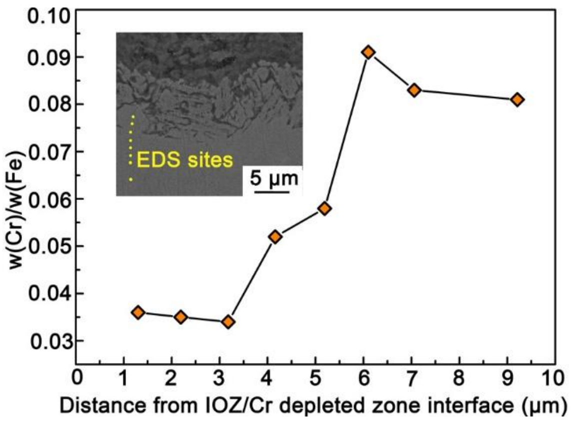

Supplementary Figure 2 | Evolution of w(Cr)/w(Fe) from IOZ/Cr depleted zone interface to Cr depleted zone/matrix interface. The value of w(Cr)/w(Fe) increases gradually from the IOZ/Cr-depleted zone interface to the Cr-depleted zone/matrix interface.

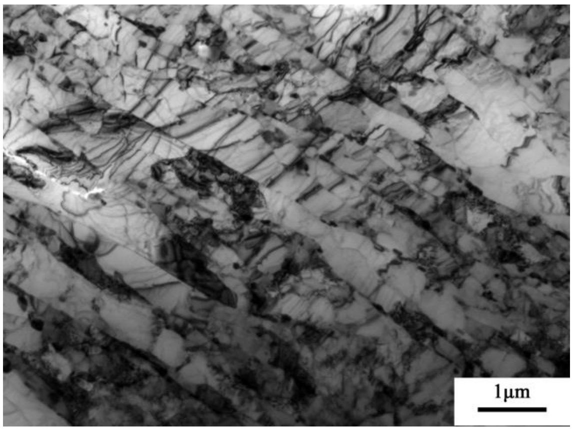

Supplementary Figure 3 | The microstructure of the investigated T91 steel before oxidation. After normalized at 1323 K and tempered at 1033 K, tempered lathy martensitic microstructure is obtained in T91 steel.

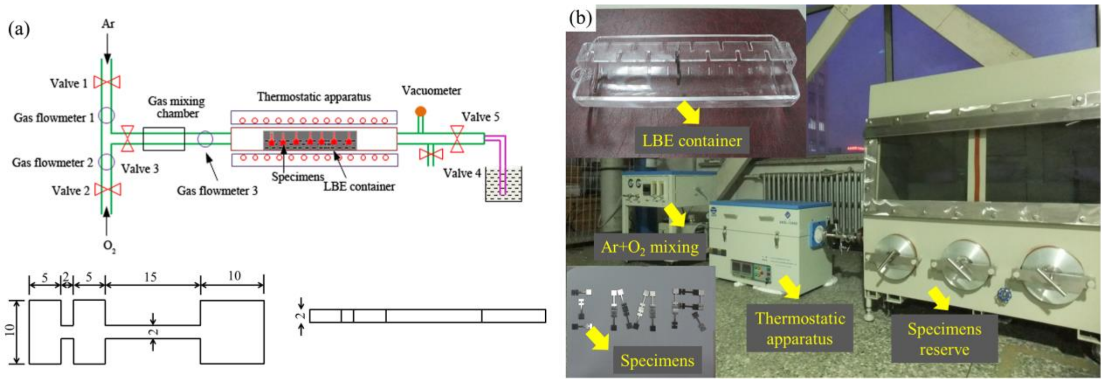

Supplementary Figure 4 │ Schematic illustration and photograph of the oxidation setup and the specimen for the oxidation experiment. (a) Schematic illustration of the oxidation setup and the specimen for oxidation experiment; (b) Photo of the oxidation setup and the specimen for oxidation experiment.

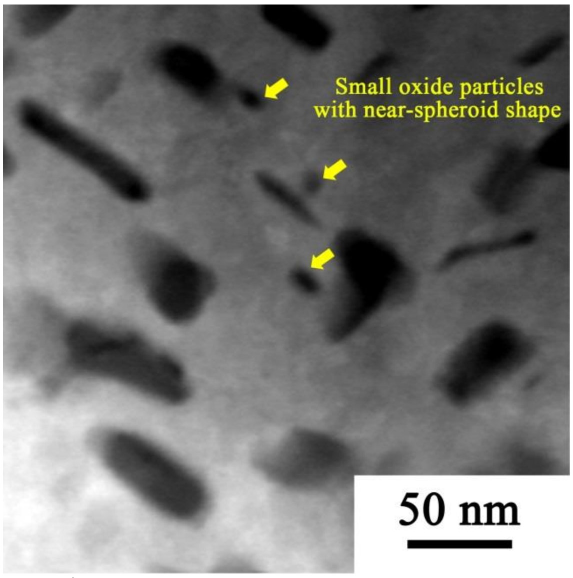

Supplementary Figure 5 | Small oxide particles with spheroid shape in the lath interior surrounded by PORs. The oxide precipitates indicated by arrows are significantly smaller than other oxide particles. In contrast to the surrounding larger oxide precipitates with square or rectangle shapes, these smaller oxide particles possess a spheroid shape.

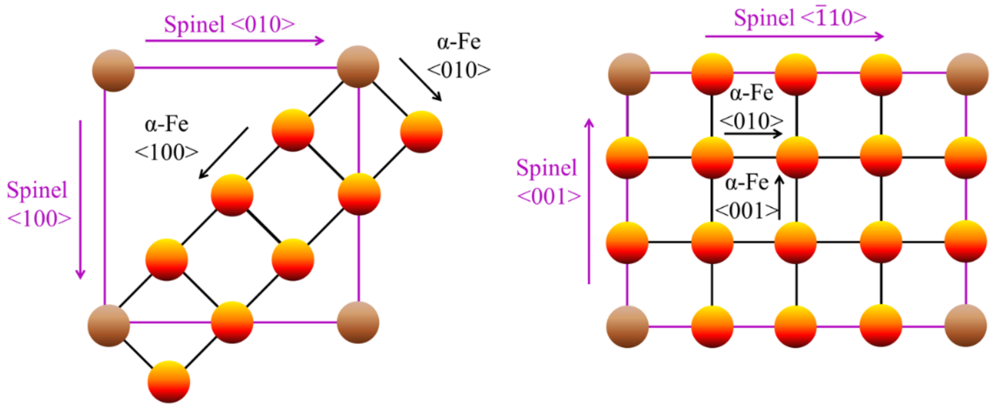

Supplementary Figure 6 | Schematic illustration of the coherent relationship between spinel oxide precipitates and the steel matrix.

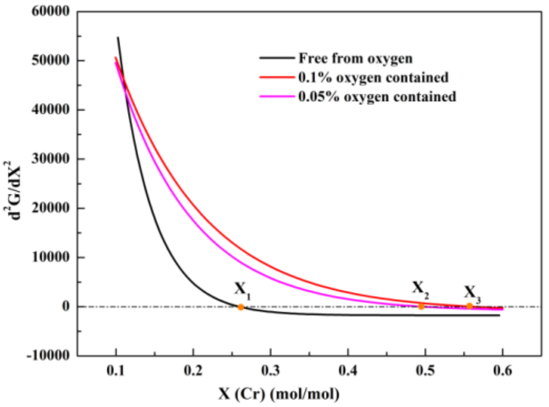

Supplementary Figure 7 │ Second derivative of Gibbs free energy of Fe-Cr-xO b.c.c. lattice with respect to mole fraction of Cr. It can be found that the critical Cr content for spinodal decomposition increases with the increasing in the oxygen concentration.

<u>SUPPLEMENTARY NOTE 1：Analysis of the SAED pattern of the oxide precipitates and the surrounding b.c.c matrix in the ODZ</u>

When we consider the space group of Fe-Cr spinel ($Fd3\overline{m}$), the diffraction spots of 00*l* reflections, where *l* is odd, should be forbidden. However, because of the second diffraction, these forbidden diffraction spots appear. In addition, the SAED patterns shown in Fig. 3c involve a set of diffraction spots of the [110] crystal zone of Fe-Cr spinel induced by the second diffraction from the diffracted electron beam of the matrix. Detail analysis of the SAED pattern in Fig. 3c is shown in Fig. 3d.

SUPPLEMENTARY NOTE 2：The morphology evolution of oxide precipitates in ODZ

An interesting finding in the ODZ is the existence of numerous nanometer oxide precipitates with two distinct characteristics: I. the regular alignment of the oxide precipitates (see Fig. 3a) and II. increase in the aspect ratio of oxide precipitates with growth time (see Figs. 3a and b).

The interfacial energy and the elastic strain energy caused by the lattice mismatch between the precipitates and the matrix make important contributions to the total free energy of a coherent system[1,2]. The morphology of a precipitate is actually determined by the balance between the strain energy and the interfacial energy. The relative importance of the elastic and interfacial energies can be evaluated by the magnitude of a dimensionless parameter, $L=\varepsilon^2 l C_{44}/\sigma$, where ε is the particle-matrix misfit, *l* is the characteristic length of a particle, C_{44} is an elastic constant, and σ is the unit area interfacial energy[2,3]. When a precipitate has a small characteristic length *l*, the interfacial energy is dominant compared with the strain energy. Therefore, a precipitate is nearly spherical to minimize the surface area and the total interfacial energy. As shown in **Supplementary Fig. 5** the precipitates indicated by arrows are significantly smaller than other oxide particles. In contrast to the surrounding larger precipitates with square or rectangle shapes, these smaller oxide particles possess a spheroid shape.

As the growth time is prolonged, the increase in the size of the oxide precipitates results in an increase in characteristic length *l* and hence the parameter L, which results in the enhancement in the elastic energy. Therefore, as the oxide particles grow to a critical size, the elastic energy becomes predominant in determining the morphology. The elastic strain around a precipitate mainly arises from coherence, i.e., the elastic strain arising from the lattice mismatch between it and the surrounding matrix. Since the lattice mismatch is crystallographically anisotropic, the resultant coherent strain is anisotropic, which determines the morphological evolution of precipitates[4,5]. The Fe-Cr spinel has an f.c.c. structure with a_{spinel}=8.379 Å, while the matrix has a b.c.c. structure with a_{Fe}=2.876 Å. According to the diffraction pattern shown in Fig. 3c, we suggest that 24-unit b.c.c Fe matrix is fitted into a unit cell of the Fe-Cr spinel (schematically shown in **Supplementary Fig. 6**) to maintain coherence/partial coherence. It is obvious that the misfit in $[100]_{spinel}$ and $[010]_{spinel}$ is $(2\sqrt{2}\times 2.876-8.379)/8.379 = -0.0292$, and the misfit in $[001]_{spinel}$ is

(3×2.876-8.379)/8.379 = 0.0297. That is to say, there is a tensile strain in the [001] direction of the oxide spinel, while a compressive strain is added to the [100] and [010] directions.

According to [6], when a tensile strain is applied to a precipitate, the precipitate is elongated along the applied tensile strain. On the contrary, a compressive strain would lead to the elongation of the particles perpendicular to the applied strain. Therefore, the Fe-Cr oxide spinel particles would grow faster along the [001] direction than along the [100] and [010] direction. The unequal growth rates in different crystallographic directions result in the rectangle shape of the oxide particles shown in Figs. 3a and b. Given the crystal symmetry of the spinel structure and the b.c.c matrix, these oblong-shaped oxide particles are distributed in two perpendicular directions. According to [2, 3], the equilibrium shape of a particle gradually evolves from four-fold symmetric shapes to a two-fold symmetric shape as the effect of the elastic energy increases. Therefore, as the particles grow, their aspect ratios gradually increase, as shown in Figs 3a and b.

SUPPLEMENTARY NOTE 3：Thermodynamic calculation of the Gibbs free energy of Fe-Cr-xO b.c.c. lattice with respect to mole fraction of Cr

The calculated thermodynamic curves in **Supplementary Fig. 7** demonstrate that the dissolved oxygen narrows the spinodal interval of the Fe-Cr alloy, i.e. the critical Cr content for spinodal decomposition increases. It is well known that the critical Cr content for spinodal decomposition in Fe-Cr alloy is around 20 wt.% at 823 K [7]. As stated in the paper, the Cr content in the lath interior is lower than 2 wt.% which is far below the critical composition. Thus, spinodal decomposition cannot occur in our experiment.

Reference

1. Li, D. & Chen, L. Computer simulation of stress-oriented nucleation and growth of θ′ precipitates in Al–Cu alloys. *Acta Mater* **46**, 2573-2585 (1998).

2. Thornton, K., Akaiwa, N. & Voorhees, P. W. Large-scale simulations of Ostwald ripening in elastically stressed solids: I. Development of microstructure. *Acta Mater* **52**, 1353-1364 (2004).

3. Thompson, M, Su, C. & Voorhees, P. The equilibrium shape of a misfitting precipitate. Acta metall, mater **42**, 2107-2122 (1994).

4. Su, C. H. & Voorhees, P. W. The dynamics of precipitate evolution in elastically stressed solids.2. Particle alignment. *Acta Mater* **44**, 2001-2016 (1996).

5. Vaithyanathan, V. & Chen, L. Coarsening of ordered intermetallic precipitates with coherency stress. *Acta Mater* **50**, 4061-4073 (2002).

6. Li, D. Y & Chen, L. Q. Shape evolution and splitting of coherent particles under applied stresses. *Acta Mater* **47**, 247-257 (1998).

7. Raghavan, V. Cr-Fe-Si (chromium-iron-silicon). *J Phase Equilib* **24**, 265-266 (2003).
